# Therapeutic effects of anti-CD154 antibody in cynomolgus monkeys with advanced rheumatoid arthritis

**DOI:** 10.1038/s41598-018-20566-z

**Published:** 2018-02-01

**Authors:** Eun Wha Choi, Kyo Won Lee, Hyojun Park, Hwajung Kim, Jong Hyun Lee, Ji Woo Song, Jehoon Yang, Yeongbeen Kwon, Tae Min Kim, Jae Berm Park, Sungjoo Kim

**Affiliations:** 1 Department of Veterinary Clinical Pathology, College of Veterinary Medicine & Institute of Veterinary Science, Kangwon National University, 1 Kangwondaehak-gil, Chuncheon-si, Gangwon-do, 24341 Republic of Korea; 2 Department of Surgery, Division of Transplantation, Samsung Medical Center, Sungkyunkwan University School of Medicine, 81 Irwon-ro, Gangnam-gu, Seoul, 06351 Republic of Korea; 3 Department of Surgery, Seoul National University College of Medicine, 101 Daehak-ro, Jongno-gu, Seoul, 03080 Republic of Korea; 4 Transplantation Research Center, Samsung Biomedical Research Institute, Samsung Medical Center, 81 Irwon-ro, Gangnam-gu, Seoul, 06351 Republic of Korea; 5 Laboratory Animal Research Center, Samsung Biomedical Research Institute, Samsung Medical Center, 81 Irwon-ro, Gangnam-gu, Seoul, 06351 Republic of Korea; 6 Department of Health Science and Technology, Samsung Advanced Institute for Health Sciences & Technology (SAIHST), Sungkyunkwan University, 81 Irwon-ro, Gangnam-gu, Seoul, 06351 Republic of Korea; 7 Graduate School of International Agricultural Technology and Institute of Green-Bio Science and Technology, Seoul National University, Pyeongchang, Gangwon-do, 25354 South Korea

## Abstract

Rheumatoid arthritis is one major chronic inflammatory systemic autoimmune disease. The CD154-CD40 interactions play a critical role in the regulation of immune responses and the maintenance of autoimmunity. Therefore, we aimed to determine whether anti-CD154 antibody treatment show positive effects on immunomodulation and clinical improvement of sustained severe rheumatoid arthritis in cynomolgus monkeys. Arthritis was induced using chicken type II collagen (CII) and arthritic monkey were divided into control and anti-CD154 treatment groups based on their concentrations of anti-CII antibodies on week 7 post-immunization. Blood and tissue samples were collected on week 16 post-immunization. Anti-CD154 antibody treatment improved arthritis and movement, and significantly decreased the numbers of proliferating B cells and the serum levels of anti-type II collagen antibody and sCD154 compared with non-treatment group. Further anti-CD154 antibody treatment significantly decreased the percentage of CD4+ cells and the ratio of CD4+ to CD8+ T cells and significantly increased the percentage of CD8+ cells and effector memory CD8+ cells in peripheral blood. We have shown for the first time in a nonhuman primate model of RA that CD154 blockade has beneficial effects. This study might be valuable as preclinical data of CD154 blockade in nonhuman primate models of severe rheumatoid arthritis.

## Introduction

Rheumatoid arthritis (RA) is one of the major chronic inflammatory systemic autoimmune diseases^1,2^. Collagen-induced arthritis rodent models have been extensively used in RA research^3–5^. However, it is preferable to study arthritis in nonhuman primates because they share many similar immunological and pathological features with humans^6,7^.

Furthermore, monoclonal antibodies to certain proteins are shared by humans and monkeys and treatment using these antibodies can be carried out in monkey models with greater predictive value of efficacy, side effects, and the pathological roles of the proteins in humans than using rodent models^6^.

CD154 contributes to the acceleration of autoimmune disease^8–11^. CD154 triggers numerous inflammatory functions in various cell types by interacting with CD40; the CD154-CD40 interaction mediates T-cell priming, B cell–dependent Ig class switching, germinal center formation, cell proliferation, release of proinflammatory cytokines, and upregulation of adhesion molecules and costimulatory molecules^12–14^. It was reported that patients with systemic lupus erythematosus (SLE), RA, and Sjögren's disease showed increased levels of soluble CD154 associated with disease activity^15–18^. Thus, some preclinical and clinical studies evaluating the use of anti-CD154 antibody for autoimmune diseases have been conducted.

Anti-CD154 antibody treatment prior to disease onset prolonged survival, prevented proteinuria, decreased levels of anti-dsDNA antibodies, and ameliorated glomerulonephritis in murine systemic lupus erythematosus models such as (NZB × NZW) F1 and (SWR × NZB) F1 mice^19,20^. Furthermore, anti-CD154 antibody treatment after disease onset also delayed disease progression and reversed proteinuria in spite of ongoing immune complex glomerulonephritis^19,20^.

However, a clinical study investigating the use of anti-CD154 antibody for the treatment of SLE was terminated earlier than expected because of thromboembolic events, even though anti-CD154 antibody treatment showed good clinical responses in some SLE patients, with decreased anti-dsDNA antibodies, increased C3 concentration, and decreased hematuria^21–23^.

Anti-mouse CD154 antibody treatment prior to induction of collagen-induced arthritis in mice significantly decreased serum type II collagen antibodies and ameliorated symptoms such as joint inflammation, cartilage damage, and bone erosion^24^. Anti-mouse CD154 antibody treatment in the K/BxN arthritis mouse model also showed preventive effects, but had no therapeutic effect after clinical onset^25^.

Anti-(human) CD154 antibody treatment after arthritis onset has not been studied in a monkey collagen-induced arthritis model. In this study we evaluated the therapeutic effect of anti-CD154 antibody on an established collagen-induced arthritis monkey model by monitoring the anti-type II collagen antibody concentration, clinical symptoms, clinicopathological changes, and immune cell population changes.

## Results

### Anti-CD154 antibody treatment reduced the clinical signs of arthritis.

**Figure 1:**
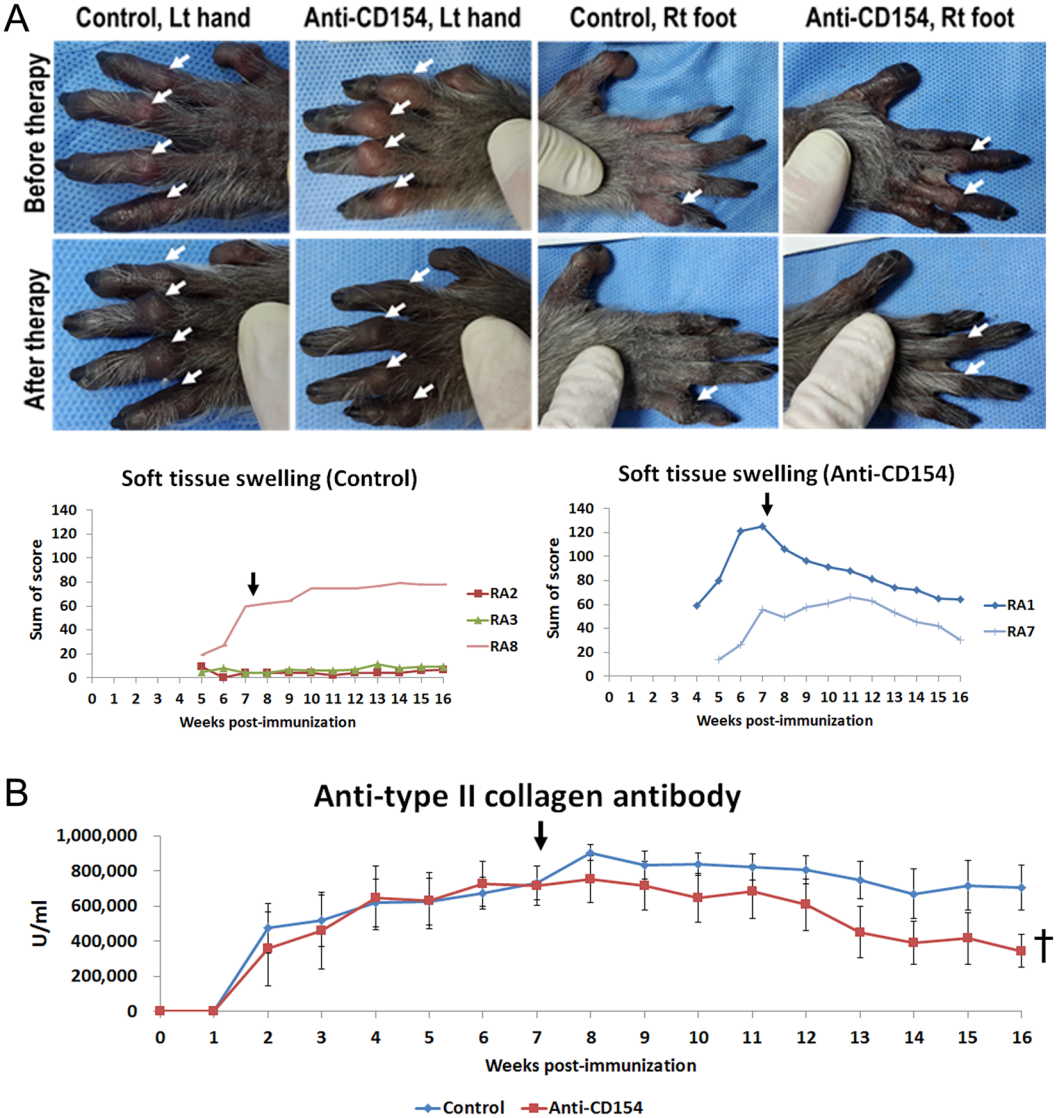
Arthritis scores and serum levels of anti-type II collagen antibody. (**A**) Representative images of the paws and scores for soft tissue swelling; left forepaws of RA8 (control group) and RA1 (anti-CD154 group) and right hindfeet of RA3 (control group) and RA7 (anti-CD154 group) before therapy (7 weeks after type II collagen immunization) and after therapy (16 weeks after immunization) are presented. Proximal interphalangeal joint swelling is marked (white arrow). After anti-CD154 antibody treatment, the sum of soft tissue swelling scores decreased in the anti-CD154 group. The sum of soft tissue swelling scores from each group is shown as a graph. The time point of primary treatment is marked by the black arrow. (**B**) Serum levels of anti-type II collagen antibody. Data are expressed as the mean ± SEM (n = 4 per group). The paired t-test (†) was used to compare means from two related samples (before vs. after therapy). “^†^”Indicates significant differences (p < 0.05). Control: control group; Anti-CD154: anti-CD154 treatment group; Lt hand: left forepaw; Rt foot: right hindfoot.

Five of eight monkeys developed soft tissue swelling in joints. Three (RA1, RA7, and RA8) of these five monkeys showed severe soft tissue swelling in proximal interphalangeal joints. The other three monkeys did not show any joint swelling, but did show joint stiffness (RA4, RA5, and RA6). After treatment with anti-CD154 antibody, the sum of soft tissue swelling scores decreased in the anti-CD154 group (RA1 and RA7) but not in the control group (RA2, RA3, and RA8) (Fig. 1A); since soft tissue swelling was not observed in all monkeys (small sample number), statistical significance was not obtained. However, after anti-CD154 treatment, the anti-CD154 group showed a decrease in soft tissue swelling score after treatment in both affected monkeys, and the untreated control group had an increased score in all three affected individuals.

### Anti-CD154 antibody treatment significantly decreased the serum levels of anti-type II collagen antibody.

Serum levels of anti-CII antibodies were significantly lower after therapy (week 16 post-immunization) than before therapy (week 7 post-immunization) in the anti-CD154 group. In contrast, this change was not observed in the control group (Fig. 1B).

### Some side effects were observed in the anti-CD154 treatment group.

**Figure 2:**
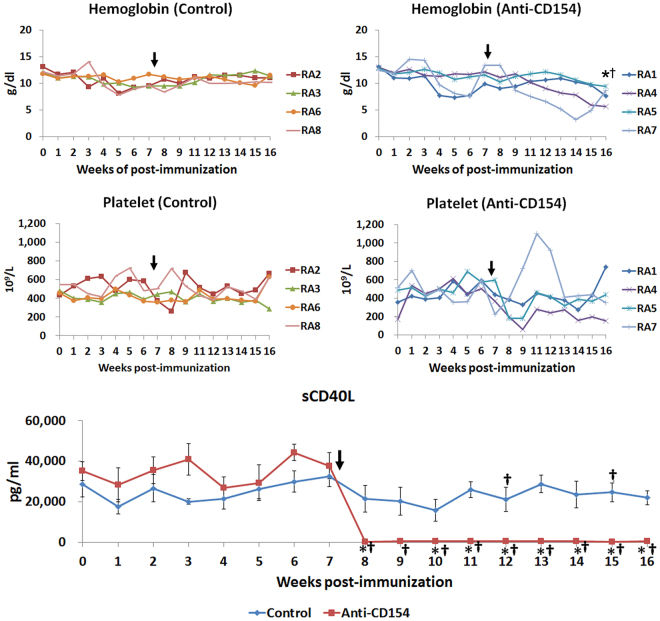
Comparison of hemoglobin level, platelet count, and soluble CD40L level, in cynomolgus monkeys with collagen-induced arthritis. Graphs of hemoglobin levels, platelet counts, and soluble CD40L levels. The time point of primary treatment is marked by the black arrow. Soluble CD40L levels are expressed as the mean ± SEM (n = 4 per group). The means of groups at each time points were compared using student t-tests. The paired t-test (†) was used to compare means from two related samples (before vs. after therapy). “*” and “^†^”Indicate significant differences (*p* < 0.05). Control: control group; Anti-CD154: anti-CD154 treatment group; sCD40L: soluble CD40 ligand.

After treatment with anti-CD154 antibody, the concentration of hemoglobin significantly decreased in the anti-CD154 group (before treatment, week 7 post immunization: 11.78 ± 1.27 g/dl and after treatment, week 16 post immunization: 7.84 ± 0.83 g/dl), but not in the control group (week 7 post immunization: 10.08 ± 0.54 g/dl and after treatment, week 16 post immunization: 11.03 ± 0.30 g/dl, Fig. 2). Further, the mean concentration of hemoglobin in the anti-CD154 group was lower than the lower limit of the reference interval of platelets (reference interval of hemoglobin: 10.10–15.45 g/dl, calculated from a previous study^26^).

Platelet number was markedly decreased due to platelet clumps in RA4 and RA5, especially during the first 2 weeks after anti-CD154 antibody therapy, and platelet numbers of RA 4 and 5 were lower than the lower limit of the reference interval of platelets (215.6–600.6 × 10^9^/L)^26^ (platelet numbers of RA 4: before treatment, week 7 post-immunization: 364 × 10^9^/L → after treatment, week 8 post-immunization: 194 × 10^9^/L and week 9 post-immunization: 57.8 × 10^9^/L and RA 5: before treatment, week 7: 596 × 10^9^/L → after treatment, week 8 post-immunization: 180 × 10^9^/L, week 9 post-immunization: 180 × 10^9^/L) (Fig. 2).

### Anti-CD154 antibody treatment significantly decreased the serum levels of sCD40L (sCD154).

Serum levels of sCD40L at week 6 post-immunization were significantly increased compared with those at week 0. After therapy, serum levels of sCD40L were significantly decreased in the anti-CD154 group, as expected. Serum levels of sCD40L at weeks 8–16 post-immunization decreased significantly compared with week 7 (before therapy) in the anti-CD154 group, and serum levels of sCD40L at weeks 8 and 10–16 post-immunization in the anti-CD154 group were decreased significantly compared with the control group (Fig. 2).

### Anti-CD154 antibody treatment significantly decreased the percentage of CD4+ cells and the ratio of CD4+ to CD8+ T cells and significantly increased the percentage of CD8+ cells and effector memory CD8+ cells in peripheral blood.

**Figure 3:**
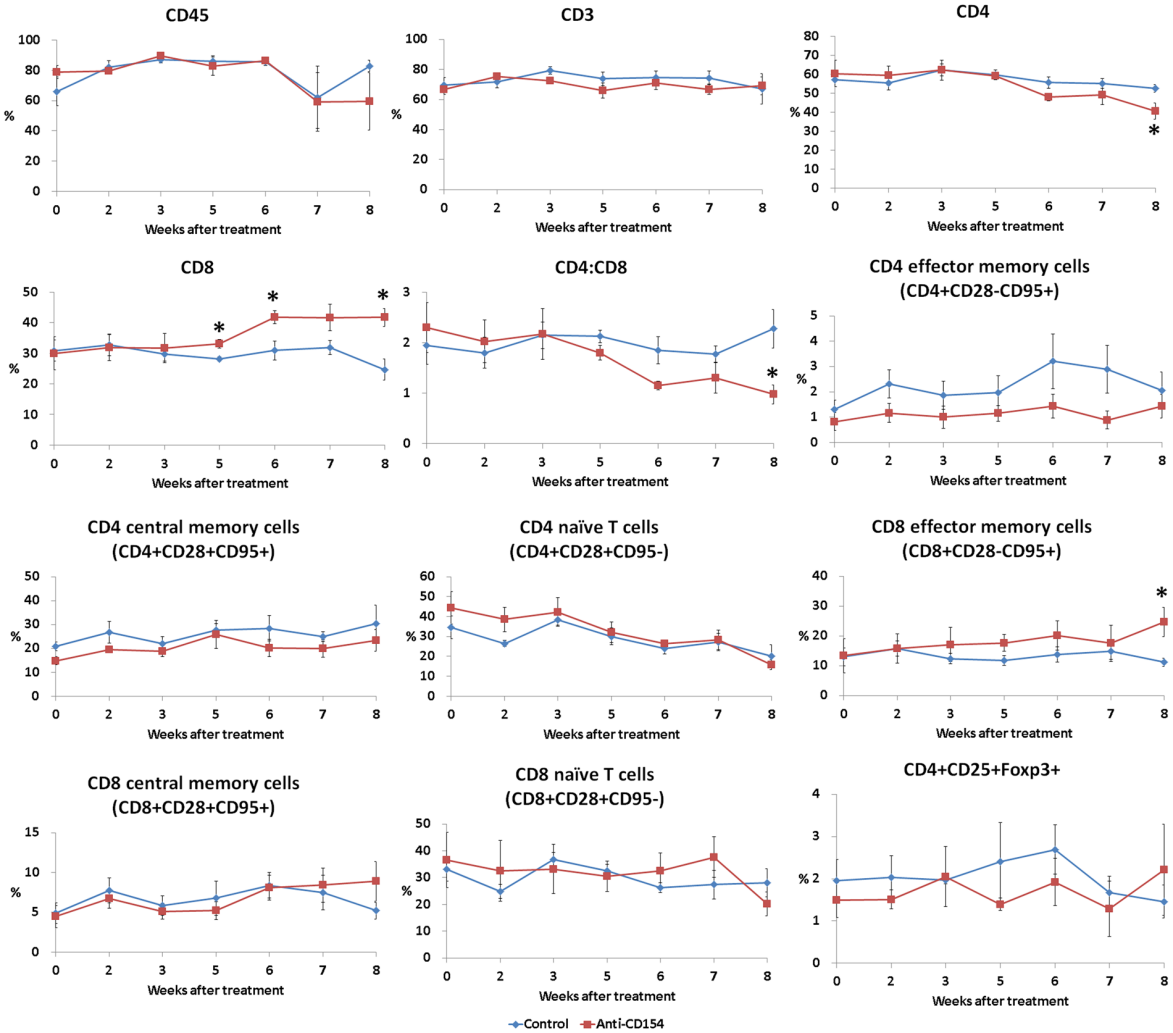
Comparison of T-cell subsets. Flow cytometric analysis of T-cell subset was performed using blood samples collected before treatment and at 2, 3, 5, 6, 7, and 8 weeks after treatment. For analysis of T-cell populations peripheral blood mononuclear cells (PBMCs) were stained with antibodies against CD45, CD3, CD4, CD8, CD28, and CD95. For analysis of regulatory T cells, PBMCs were stained with antibodies against CD45, CD4, CD25, and FoxP3. Data are expressed as the mean ± SEM (n = 4 per group). The means of groups at each time points were compared using student t-tests. *Significant (*p* < 0.05) differences from the control group are indicated.

**Figure 4:**
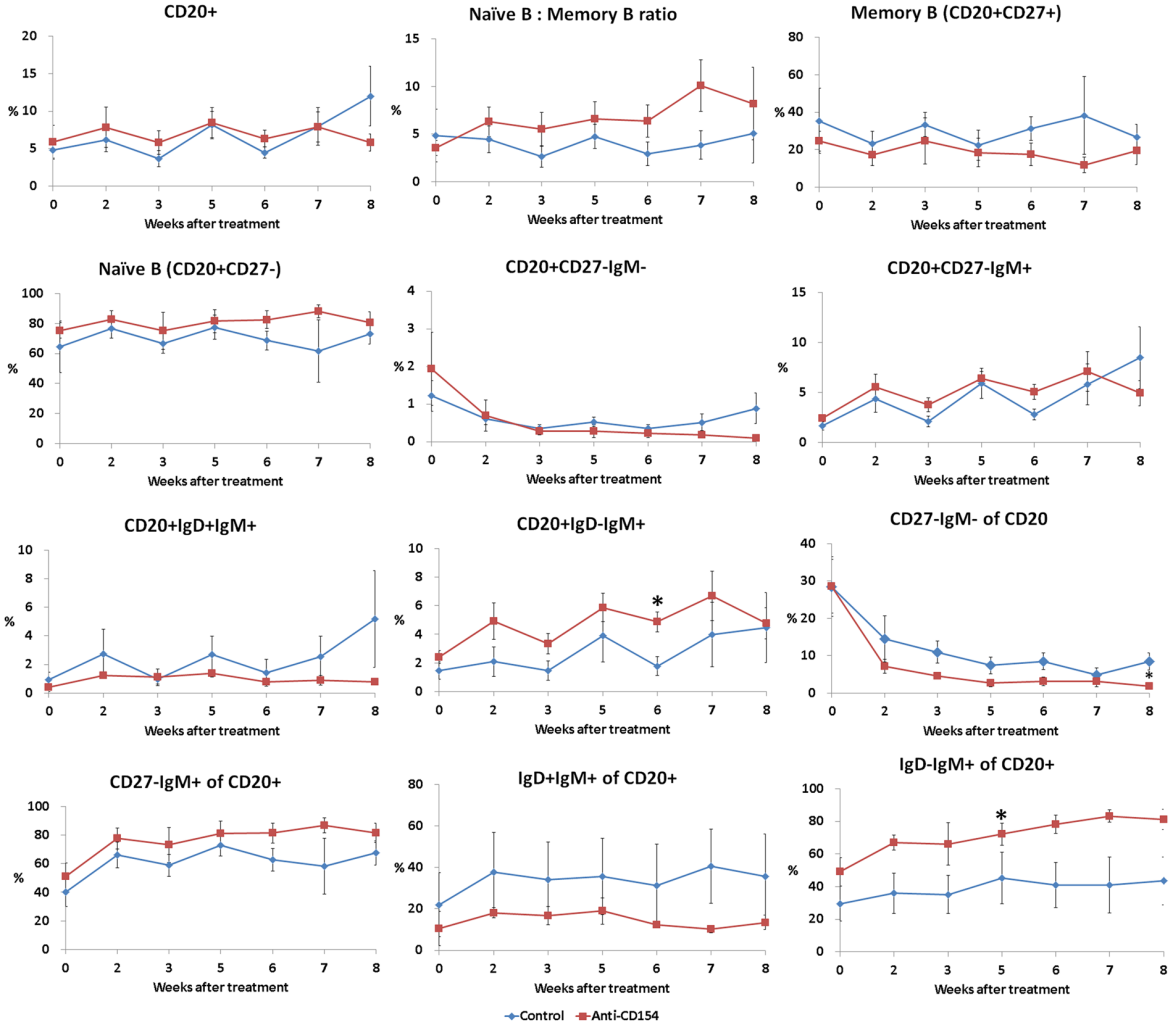
Comparison of B-cell subsets. Flow cytometric analysis for B-cell subsets was performed using blood samples collected before treatment and at 2, 3, 5, 6, 7, and 8 weeks after treatment. PBMCs were stained with antibodies against CD45, CD3, CD20, CD27, CD21, IgD, and IgM. Data are expressed as the mean ± SEM (n = 4 per group). The means of groups at each time points were compared using student t-tests. *Significant (p < 0.05) differences from the control group are indicated.

A representative gating scheme of the T cell subset, Treg cells and B cell subset in the peripheral blood are shown in the ‘Supplementary Figure 1’. The percentage of CD4+ T cells and the ratio of CD4+ to CD8+ T cells were significantly decreased in the anti-CD154 group compared with the control group at 8 weeks after treatment (Fig. 3). The percentage of CD8+ cells was significantly increased in the anti-CD154 group at 5, 6, and 8 weeks after treatment and the percentage of effector memory CD8+ T cells was significantly increased in the anti-CD154 group at 8 weeks after treatment (Fig. 3). Anti-CD154 antibody treatment showed a pattern of increased ratio of naïve (CD20+CD27−) to memory (CD20+CD27+) B cells (Fig. 4); the ratios of naïve to memory B cells in the control and anti-CD154 groups were 4.83 ± 2.78 and 3.55 ± 0.79, respectively, before treatment and 3.85 ± 1.51 and 10.09 ± 2.7 at 7 weeks after treatment.

### After treatment, lesions did not progress in anti-CD154 antibody treatment group on radiographic examination.

**Figure 5:**
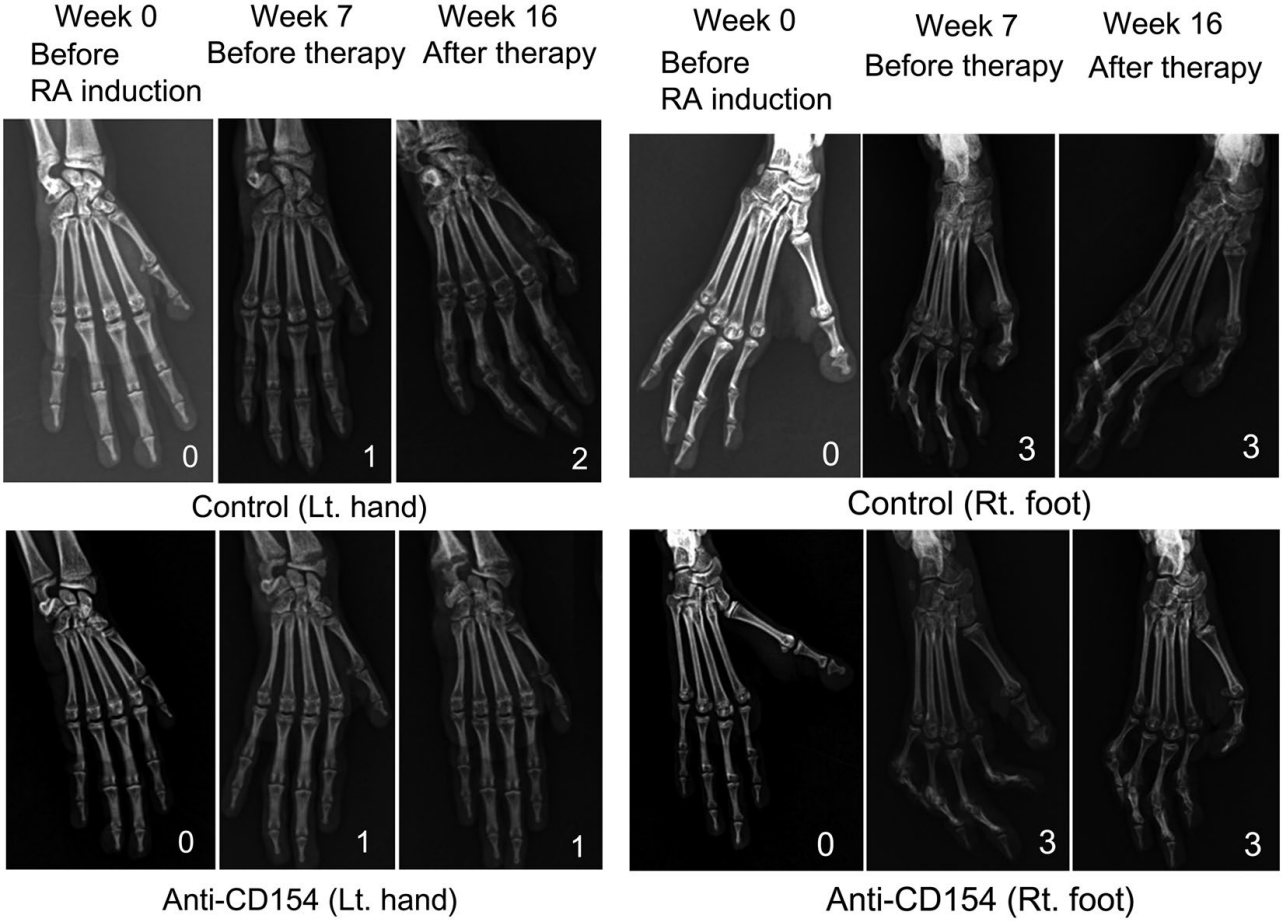
Radiographs of the forepaws or hind feet. Radiographic grading of the four limbs was carried out as follows: 0: no change, 1: disappearance of the articular cavity and progression of obscurity of the epiphysis; 2: increased number of joints lacking articular cavities, closure of the epiphysis, unclear boundary between bones, and progression of obscurity of the surroundings; 3: further advancement of these changes and bones are twisted. Radiographs of the left forepaws of RA8 (control group) and RA7 (anti-CD154 group) and the right hind feet of RA8 (control group) and RA1 (anti-CD154 group) before therapy (7 weeks after type II collagen immunization) and after therapy (16 weeks after immunization) are presented. Control: control group; Anti-CD154: anti-CD154 treatment group.

Obscurity of the epiphysis and the surroundings progressed further in RA 8 (a severely arthritic monkey in the control group), but did not progress in RA 1 and RA7 (severely arthritic monkeys in the anti-CD154 group) after treatment (Fig. 5, left forepaws). No improvement was observed in the joints that had progressed to grade 3 on X-ray (Fig. 5, right hind feet).

### Anti-CD154 antibody treatment resulted in a reduction in cartilage damage and CD154-positive cells of the arthritic monkeys.

**Figure 6:**
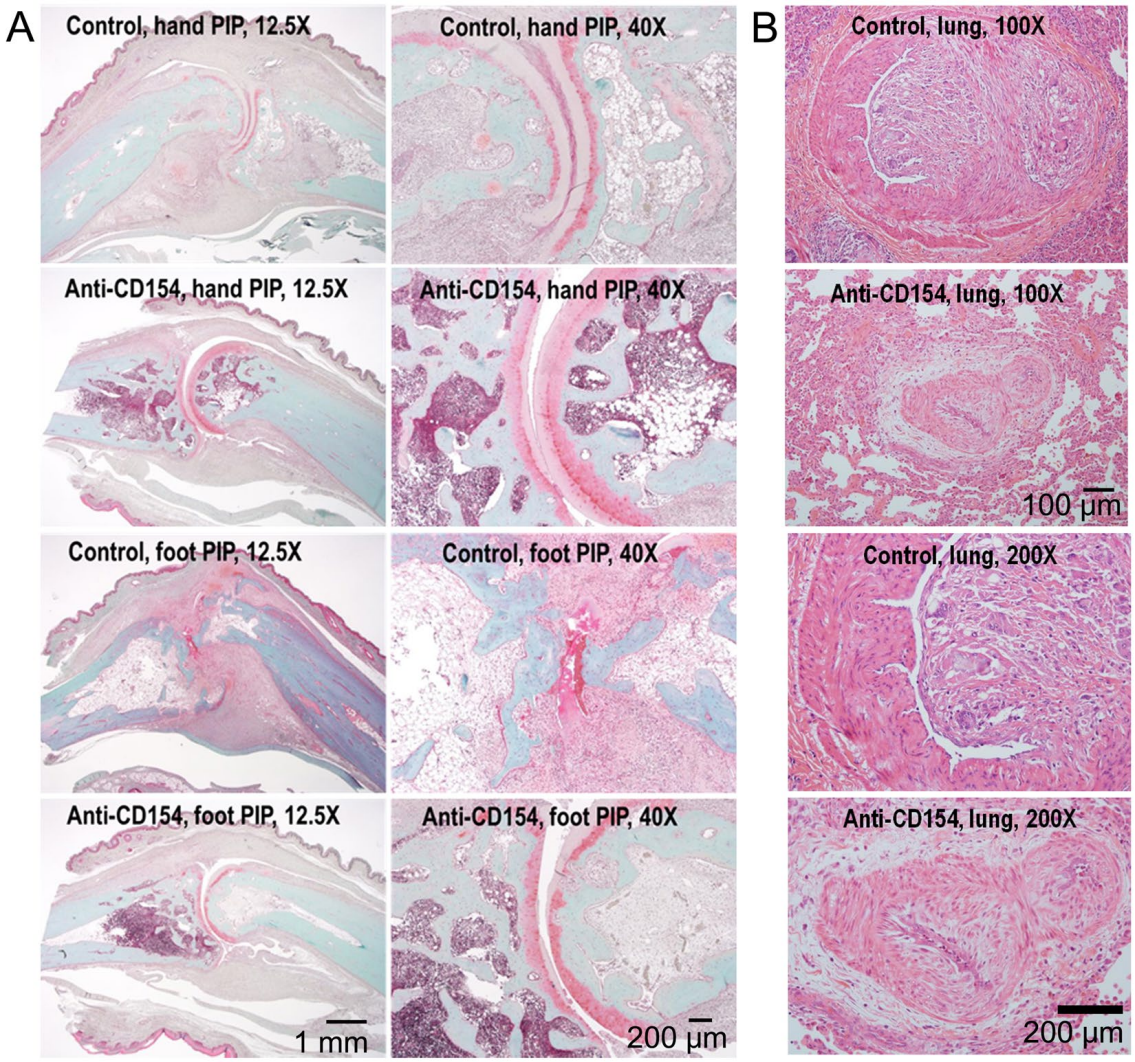
Histopathology of the proximal interphalangeal joints and lungs from arthritic monkeys. (**A**) Articular cartilage sections were stained with Safranin O. Proximal interphalangeal joints from the forepaws of RA8 (control group) and RA7 (anti-CD154 group) and from the hind feet of RA8 (control group) and RA1 (anti-CD154 group) are presented (Original magnification: 12.5x and 40x). Treatment with anti-CD154 antibody resulted in a reduction in cartilage damage of the arthritic monkeys that had a high score for soft tissue swelling. (**B**) Lung tissue sections were stained with H&E. Lung tissues from RA8 (control) and RA7 (anti-CD154) are presented (Original magnification: 100x and 200x). Irregular thickening of the arterial intima layer was observed in lungs from the monkeys in both the anti-CD154 group and the control group. Control: control group; Anti-CD154: anti-CD154 treatment group; hand: forepaw; foot: hind foot; PIP: proximal interphalangeal joints.

Treatment with anti-CD154 antibody resulted in a reduction in cartilage damage of the arthritic monkeys that had a high score for STS (Fig. 6A). Irregular thickening of the arterial intima layer was occasionally observed in lungs from monkeys in the anti-CD154 group as well as the control group. Images of very severe thickening of the arterial intima layer in lungs (RA8 control and RA7 anti-CD154) are presented in Fig. 6B.

**Figure 7:**
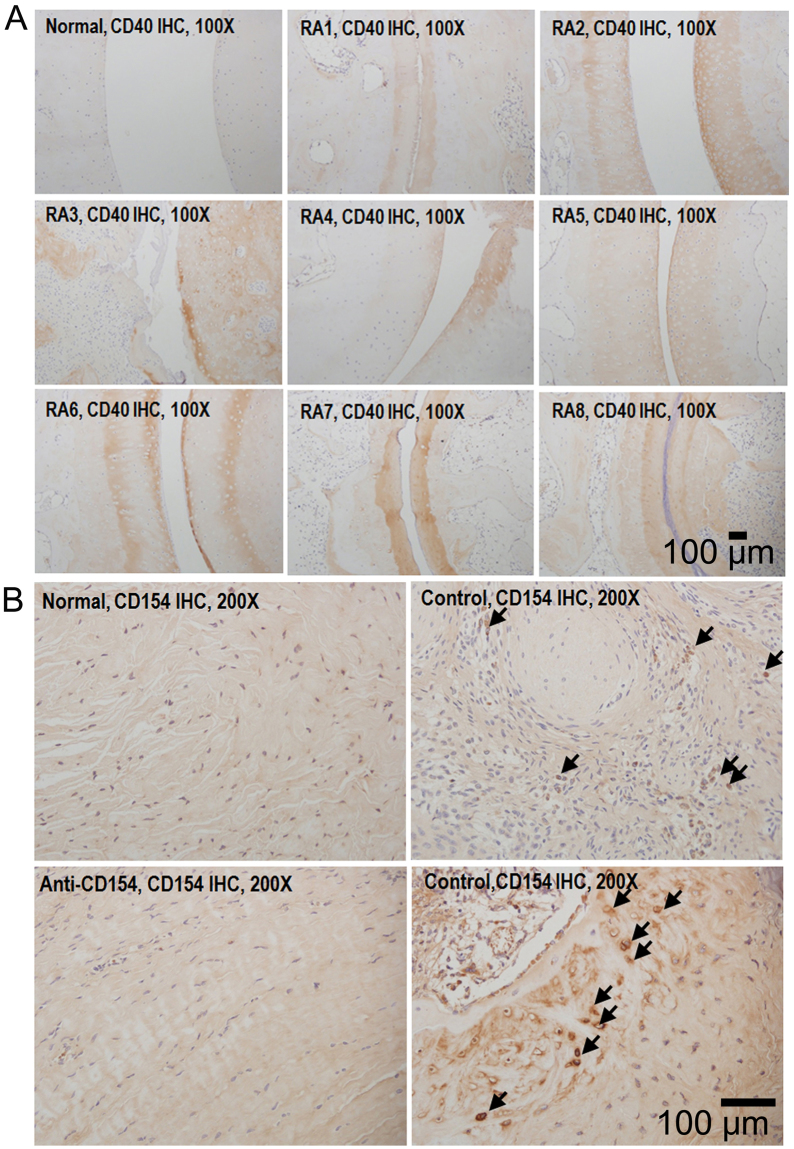
Immunohistochemical analysis of CD40 and CD154 expression in the joints. Proximal interphalangeal joint sections from arthritic monkeys were stained for CD40 and CD154. Proximal interphalangeal joint sections from normal monkeys were used as a normal control. (**A**) CD40 was expressed on the cartilage of cynomolgus monkeys with induced arthritis (original magnification: 100x). (**B**) CD154-positive cells were observed in the synovia from the non-treated control group (original magnification: 200x). Normal: healthy cynomolgus monkey; RA: cynomolgus monkey with collagen-induced arthritis; control: control group; anti-CD154: anti-CD154 treatment group.

CD40 was also expressed in the cartilage of all monkeys with induced arthritis, but not in healthy controls in which RA was not induced (Fig. 7A). CD154-positive cells were observed in synovia of non-treated control monkeys but were rare in synovia from monkeys treated with anti-CD154 antibody (Fig. 7B).

### Anti-CD154 antibody treatment significantly decreased the numbers of proliferating B cells in the spleen and lymph node.

**Figure 8:**
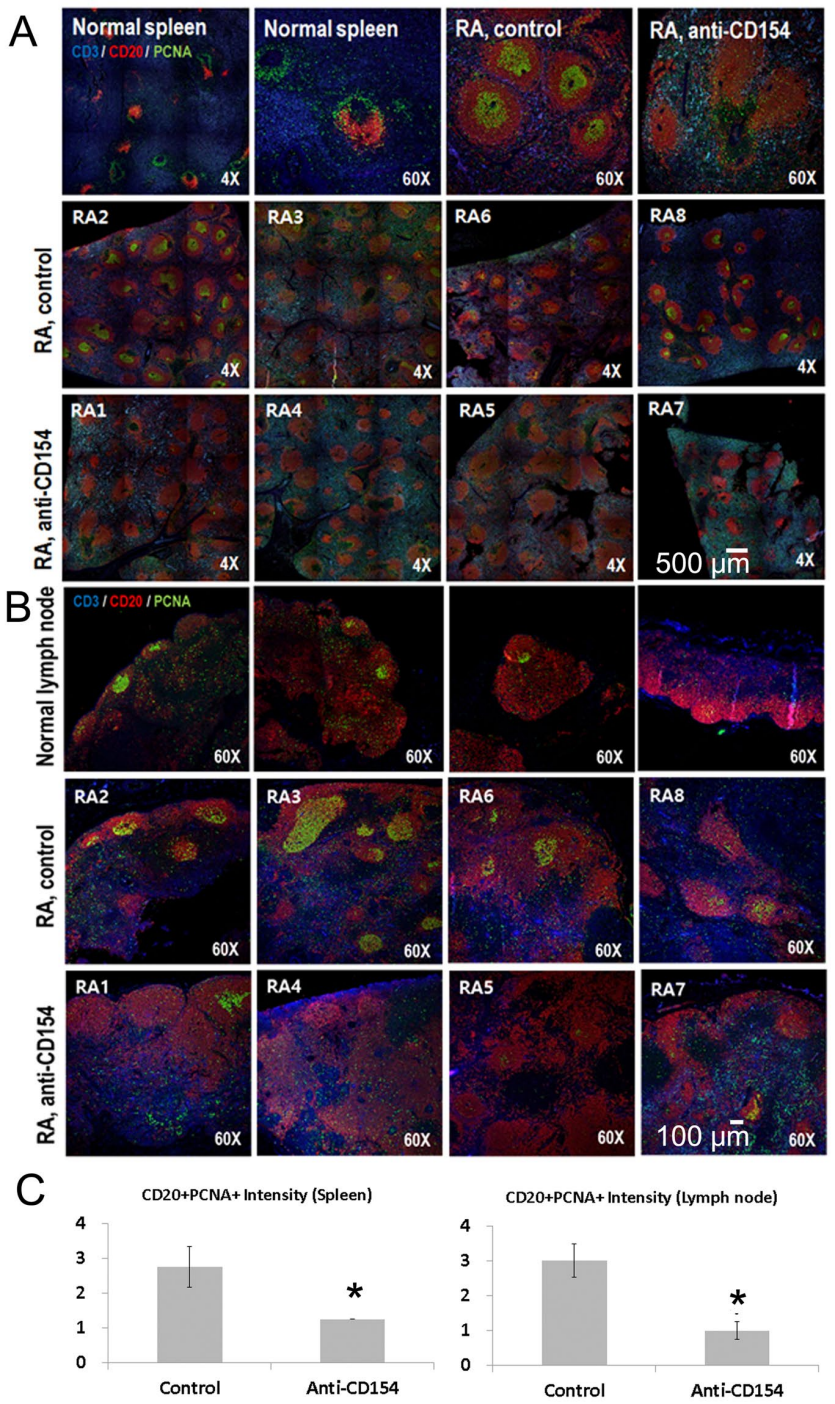
Triple immunohistochemistry for expression of CD3, CD20, and proliferating cell nuclear antigen in spleen and lymph nodes. (**A**) Spleen or (**B**) lymph node sections from arthritic monkeys were triple-stained for CD3 (blue), CD20 (red), and PCNA (green) and analyzed by confocal microscopy. The three panels were merged. Colocalization of CD20 (red) with PCNA (green) resulted in a yellowish-green color. Stained sections were analyzed using a laser scanning confocal microscope (LSM 780). (**C**) CD20+PCNA+ intensity scores. The intensity of fluorescence was graded on a scale of 1 (weak) to 4 (strong fluorescence intensity); n = 4 per group. The means of groups at each time points were compared using student t-tests. *Significant (*p* < 0.05) differences from the control group are indicated. Normal: healthy cynomolgus monkey; RA: cynomolgus monkey with collagen-induced arthritis; control: control group; anti-CD154: anti-CD154 treatment group.

Expression of CD3, CD20, and proliferating cell nuclear antigen (PCNA) in spleen and lymph node was evaluated. Spleen or lymph node sections from cynomolgus monkeys were triple-stained for CD3 (blue), CD20 (red), and PCNA (green) and analyzed by confocal microscopy. The three panels were merged. Colocalization of CD20 (red) with PCNA (green) resulted in a yellowish-green color. Enhanced proliferation of B cells was observed in the RA control group and the number of proliferating B cells (CD20+PCNA+) was significantly lower in the anti-CD154 group than in the control group (Fig. 8A–C).

### Anti-CD154 antibody treatment improved the movement.

After anti-CD154 treatment, movement was improved in RA1 and RA7 (anti-CD154 treatment group), but not in RA8 (control group) (Supplementary video file).

## Discussion

Anti-CD154 antibody treatment reduced the intensity scores and incidence of soft tissue swelling, improved movement, and significantly decreased the number of proliferating B cells and the levels of anti-type II collagen antibody in cynomolgus monkeys with advanced rheumatoid arthritis. The expression of CD154 on activated T cells and serum levels of sCD154 are increased in autoimmune diseases, and increased signaling via CD40 on B cells, monocytes, and dendritic cells promotes the production of autoantibodies and pro-inflammatory cytokines^18^.

Further immunohistochemical study revealed that CD40 was expressed on chondrocytes in patients with RA but not in patients with osteoarthritis^27^. It was also suggested that the CD40-CD154 interaction increases the expression of inflammatory cytokines such as TNF-α, IL-6, and matrix metalloproteinase in chondrocytes and contributes to degradation of the cartilage in RA^27^. In this study, CD40 was expressed on the cartilage in all of the monkeys with induced arthritis, but not in healthy controls in which RA was not induced. CD154-positive cells were observed in the synovia of non-treated control monkeys.

Anti-CD154 antibody treatment induced modulation of the proliferating B population and the autoantibody response as well as a reduction of sCD154 levels, which seemed to protect monkeys from the clinical and pathologic effects of collagen-induced arthritis.

Further anti-CD154 antibody treatment significantly decreased the percentage of CD4+ cells and the ratio of CD4+ to CD8+ T cells and significantly increased the percentage of CD8+ cells and effector memory CD8+ cells. A high ratio of CD4+ to CD8+ T cells was observed in the blood of patients with RA^28^, and in the spleen of mice with collagen induced arthritis^29^. It was also reported that levels of terminally differentiated effector memory CD8+ T cells in peripheral blood were significantly decreased in patients with RA, which may reflect increased migration of these cells to sites of inflammation and might play a role in the pathogenesis of RA^30^.

There are a few reports on the characteristics of CD8+ T cells in rheumatoid arthritis. A previous study reported that the percentage of synovial CD8+ T cells was significantly increased in patients with RA and was inversely associated with disease activity (disease activity score based on 28 joint counts, DAS28ESR)^30^. Furthermore, the authors of that study also demonstrated that synovial effector memory CD8+ T cells had a type 2 cytotoxic T lymphocyte (Tc2)-skewed phenotype and increased IL-10 producing suppressor T cells (Ts); increased levels of Tc2 and Ts cells could be beneficial in controlling inflammation of RA^31^.

Thus the decreased ratio of CD4+ to CD8+ T cells and increased effector memory CD8+ T cells might be involved in the therapeutic mechanism of anti-CD154 antibody treatment, although further study is needed for characterization of effector memory CD8+ T cells and elucidation of its role in RA.

As described above, anti-CD154 antibody treatment improved the symptoms of rheumatoid arthritis but some side effects were observed. After anti-CD154 antibody treatment, the hemoglobin level was decreased in all monkeys and platelet numbers were markedly decreased due to platelet clumps in two of four monkeys of the anti-CD154 group, especially during the first 2 weeks after anti-CD154 antibody therapy. Platelets in clumps are not counted by the hematological analyzer and therefore lead to a falsely low count^32^. Increased intima layer thickness in pulmonary arteries was observed in both groups, especially in severely arthritic monkeys. According to a previous study on cardiovascular risk in patients with asymptomatic low-grade carotid stenosis, increased levels of CRP, IL-6, and sCD154 are linked with a high risk of atherothrombosis^33^. Thus, the severe RA condition in the control group (RA8) might induce thickening of the intima layer in pulmonary arteries. In contrast to human and nonhuman primate platelets, mouse platelets do not express FcRs^34^. Furthermore, the amount of blood that can be sampled is not sufficient to examine blood parameters serially due to the small body size of mice. Therefore, monkey models are better than mouse models for examining the side effects related to blood parameters and thromboembolism events.

Recently, modified anti-CD154 antibodies that do not activate platelets but effectively inhibit CD154-dependent immune responses have been developed^34,35^. The present study might provide valuable preclinical data for CD40-CD154 blockade in nonhuman primate models with severe RA.

We expect that treatment at an earlier stage of disease might be more effective for protecting against cartilage damage and preventing development of arthritis by reducing the formation of autoantibodies against type II collagen^19^. We have shown for the first time in a nonhuman primate model of RA that CD154 blockade has beneficial effects in the treatment of the advanced stage of RA.

## Materials and Methods

### Experimental animals

Cynomolgus monkeys were purchased from ORIENT CAM CO., LTD. (Kampong Chhnang, Cambodia) and acclimatized for 4 weeks before study commencement. The total study group comprised eight healthy female cynomolgus monkeys of approximately 3 years of age and weighing 2–3 kg. The monkeys were housed singly in stainless steel caging in a room maintained at 23 ± 3 °C (June–August: 25 ± 4 °C) with 30–70% humidity and were fed with food pellets (Primate Diet 5048, Lab Diet, St. Louis, MO, USA), vegetables, and fruits under specific pathogen-free (SPF) conditions. Drinking water was provided ad libitum.

Analgesic medication (Tridol, 2 mg/kg bid) was given based on assessment by the animal caretakers and a veterinarian. Ulcerative skin lesions that developed at the immunization sites were cleaned with saline solution and antibiotic ointment was applied (Bactroban Ointment, HANALL BIOPHARMA, Daejeon, Korea). The study was reviewed and approved by the Institutional Animal Care and Use Committee of Orient Genia (Sungnam, Korea; Approval number: ORIENT-IACUC-15077). All procedures were in compliance with Animal Welfare Act Regulations and the Guide for the Care and Use of Laboratory Animals. The facility has been audited by Korean Quarantine Inspection Agency and Ministry of Food and Drug Safety.

### Induction of collagen-induced arthritis

For the induction of collagen-induced arthritis, chicken type II collagen (4 mg/ml, CII; Sigma-Aldrich, St. Louis, MO, USA) was dissolved overnight in 0.1 M acetic acid (Sigma-Aldrich) at 4 °C and emulsified with complete Freund’s adjuvant (vol:vol = 1:1; F5881, Sigma-Aldrich) using an electronic homogenizer (30,000 rpm, 3 min, Polytron PT3100D; Kinematica, Bohemia, NY, USA) in an ice-water bath. One milliliter of the emulsion (2 mg CII/monkey) was injected intradermally at 10–20 sites over the back and base of the tail of the monkeys for primary immunization. Three weeks later, monkeys were given a subcutaneous booster injection (10–20 injections on the back) of 2 mg CII emulsified in incomplete Freund’s adjuvant (F5506; Sigma-Aldrich).

### Clinical assessment of arthritis

Once a week, soft tissue swelling (STS) of each joint was scored on a graded scale from 0 (no STS) to 5 (severe STS). Swelling was analyzed by measuring the volume of the hands and feet by water immersion. The number of all joints with STS was counted. All joints of the fingers, toes, elbow, knee, wrist, and ankle (total 64 joints) were investigated.

### Blood parameters

Blood samples were collected from the femoral vein once a week under ketamine sedation. Complete blood count (CELL DYN 3700, Abbott Diagnostics, Lake Forest, IL, USA) and serum chemistry analysis were conducted (HITACHI 7180 Clinical analyzer, Hitachi Chemical, Osaka, Japan); alanine transaminase (ALT), aspartate transaminase (AST), total bilirubin, blood urea nitrogen (BUN), creatinine, total protein, albumin, Na, K, Cl, and C-reactive protein (CRP) were measured.

### Radiographic examination

Radiographic projections were made once a week using the Veterinary Digital Radiographic Imaging System (LATIVET, ATLAIM Corporation, Seongnam, Korea), and radiographic grading of the four limbs was carried out according to a previous study^6^ (0: no change, 1: disappearance of the articular cavity and progression of obscurity of the epiphysis; 2: increased number of joints lacking articular cavities, closure of the epiphysis, unclear boundary between bones, and progression of obscurity of the surroundings; 3: further advancement of these changes and bones are twisted).

### Detection of anti-type II collagen antibody

CII antibodies were measured using a commercial human/monkey anti-chick type II collagen IgG antibody assay kit (Chondrex, Inc., Redmond, WA, USA) following the manufacturer’s instructions. Monkey sera collected at 1 week and at 2–16 weeks were diluted 1:1,000 and 1:40,000 respectively. The concentration calculated from the standard curve was multiplied by the dilution factor.

### Treatments

Based on serum anti-CII antibody concentration at week 7 post-immunization, monkeys were divided into two groups: control (n = 4) and anti-CD154 (intact IgG1; C10, provided by Progen Co. Ltd., Seongnam, Korea) group (n = 4). From day 55 post-immunization, monkeys in the anti-CD154 group were intravenously administered 20 mg of CD154 antibody daily for 7 days and then weekly for 8 weeks.

### Flow cytometry

Flow cytometric analysis was performed using blood samples collected before treatment (weeks 7–8 post-immunization) and at 2, 3, 5, 6, 7, and 8 weeks after treatment (weeks 9–10, 10–11, 13, 14, 15, and 16 post-immunization). All antibodies were purchased from BD Biosciences (San Jose, CA, USA). For analysis of T-cell populations, peripheral blood mononuclear cells (PBMCs) were stained with V500–conjugated anti-CD45 (BD Biosciences), PE-Cy7–conjugated anti-CD3, PerCP-Cy5.5–conjugated anti-CD4, APC-Cy7–conjugated anti-CD8, PE–conjugated anti-CD28, and APC–conjugated anti-CD95 antibodies. For analysis of B-cell populations, PBMCs were stained with V500–conjugated CD45, PE-cy7–conjugated anti-CD3, FITC–conjugated anti-CD20, V450–conjugated anti-CD27, PerCP-Cy5.5–conjugated anti-CD21, PE–conjugated anti-IgD, and APC–conjugated anti-IgM antibodies. For analysis of regulatory T cells, PBMCs were stained with V500–conjugated anti-CD45, FITC–conjugated anti-CD4, PE-Cy7–conjugated anti-CD25, and PE–conjugated anti-FoxP3 antibodies. Flow cytometric analysis was carried out using a BD LSRFORTESSA cell analyzer (BD Biosciences).

### Histopathology

Histological study was carried out at the end of the study (week 16 post-immunization). Affected interphalangeal joints were harvested from the monkeys with STS, and the third digits of the hands and feet were harvested from monkeys without STS. For histopathological examination, the proximal interphalangeal joint tissues were fixed with 10% neutral buffered formalin (Sigma-Aldrich) and subsequently decalcified with 15% EDTA pH7.4 (BIOSESANG, Sungnam, Korea) on a shaker at room temperature for 24 days. The solution was changed twice. Decalcification was considered complete when the bone tissue could easily be cut with a razor blade. Following decalcification, the tissues were dehydrated and embedded in paraffin. For histopathological assessment of damage, the paraffin-embedded tissues were sliced into 4-µm sections, deparaffinized in xylene, rehydrated in graded alcohol, and stained with hematoxylin and eosin (H&E, DAKO, Carpinteria, CA, USA) and safranin O (IHC world, Woodstock, MD, USA). The severity of cartilage damage was scored on a graded scale from 0 (no damage) to 4 (severe damage).

Macroscopic examination of pulmonary tissues included partial dissection of pulmonary and bronchial arteries. Ten histopathology sections were taken from each of the four lobes.

### Triple immunohistochemistry

Spleen and lymph node samples were fixed by immersion in 10% neutral-buffered formalin (Sigma-Aldrich), embedded in paraffin, and sectioned at 4 μm. Paraffin-embedded sections were deparaffinized in xylene, rehydrated in graded alcohol, and transferred to 0.01 M phosphate-buffered saline (PBS, pH 7.4). Subsequently, sections underwent heat-induced epitope retrieval with Tris/EDTA buffer (pH 9.0; Dako) for 5 minutes at 121 °C to reveal hidden antigen epitopes followed by cooling to room temperature. Endogenous peroxidase was blocked by incubating the slides with 3% hydrogen peroxide in PBS for 10 min at room temperature. After washing in PBS, sections were treated with 10% normal goat serum (Dako) for 1 hour at room temperature to block nonspecific binding. Subsequently, sections were incubated overnight at 4 °C with mouse anti-CD20 (DAKO, M0755, 1:500), rat anti-CD3 (Abcam, ab56313, 1:400), and rabbit anti-PCNA (Abcam, ab18197, 1:6,000) antibodies. After washing in PBS, the sections were incubated for 2 hours at room temperature with fluorescence conjugated donkey anti-mouse (Alexa Fluor® 594; Abcam, ab150108, 1:500), donkey anti-rat (Alexa Fluor® 405; Abcam, ab175670, 1:500) and donkey anti-rabbit (Alexa Fluor® 488; Abcam, ab150073, 1:500) antibodies. The slides were washed in PBS (Sigma-Aldrich) three times for 5 min each and stained sections were analyzed using a laser scanning confocal microscope (LSM 780; Carl Zeiss, Jena, Germany).

### Statistical analysis

All results are expressed as the mean ± the standard error of the mean (SEM). The means of groups were compared using student t-tests. The paired t-test was used to compare means from two related samples (before vs. after treatment). Confidence levels of 95% or higher were considered significant (*p* < 0.05). All statistical analyses were performed using SPSS version 22.0 (IBM, Armonk, NY, USA).

## Electronic supplementary material

Supplementary Figures

Supplementary video
