# Supplementary material for: Therapeutic effects of anti-CD154 antibody in cynomolgus monkeys with advanced rheumatoid arthritis
Source: Sci Rep. 2018 Feb 1;8:2135. doi: 10.1038/s41598-018-20566-z (PMC5794761; doi:10.1038/s41598-018-20566-z)
Supplement: Supplementary file 1 — Supplementary Figures [file 41598_2018_20566_MOESM1_ESM.pdf]

Supplementary information

Title:

Therapeutic effects of anti-CD154 antibody in cynomolgus monkeys with advanced rheumatoid arthritis

Authors:

Eun Wha Choi[1,*], Kyo Won Lee[2], Hyojun Park[2], Hwajung Kim[3], Jong Hyun Lee[4], Ji Woo Song[4,5], Jehoon Yang[5], Yeongbeen Kwon[6], Tae Min Kim[7], Jae Berm Park[2], Sungjoo Kim[2,4,*]

[1]Department of Veterinary Clinical Pathology, College of Veterinary Medicine & Institute of Veterinary Science, Kangwon National University, 1 Kangwondaehak-gil, Chuncheon-si, Gangwon-do, 24341, Republic of Korea

[2]Department of Surgery, Division of Transplantation, Samsung Medical Center, Sungkyunkwan University School of Medicine, 81 Irwon-ro, Gangnam-gu, Seoul, 06351, Republic of Korea

[3]Department of Surgery, Seoul National University College of Medicine, 101 Daehak-ro, Jongno-gu, Seoul, 03080, Republic of Korea.

[4]Transplantation Research Center, Samsung Biomedical Research Institute, Samsung Medical Center, 81 Irwon-ro, Gangnam-gu, Seoul, 06351, Republic of Korea

[5]Laboratory Animal Research Center, Samsung Biomedical Research Institute, Samsung Medical Center, 81 Irwon-ro, Gangnam-gu, Seoul, 06351, Republic of Korea

[6] Department of Health Science and Technology, Samsung Advanced Institute for Health Sciences & Technology (SAIHST), Sungkyunkwan University, 81 Irwon-ro, Gangnam-gu, Seoul, 06351, Republic of Korea

[7]Graduate School of International Agricultural Technology and Institute of Green-Bio Science and Technology, Seoul National University, Pyeongchang, Gangwon-do, 25354, South Korea

*Corresponding author: Eun Wha Choi, DVM, PhD, Assistant Professor

Address: Department of Veterinary Clinical Pathology, College of Veterinary Medicine, Kangwon National University, 1 Kangwondaehak-gil, Chuncheon-si, Gangwon-do 24341, Republic of Korea

Telephone: 82-33-250-8794, Fax: 82-33-259-5625

E-mail: ewchoi@kangwon.ac.kr

*Corresponding author: Sung Joo Kim, MD, PhD, Professor

Department of Surgery, Division of Transplantation, Samsung Medical Center, Sungkyunkwan University School of Medicine, 81 Irwon-ro, Gangnam-gu, Seoul 135-710, Republic of Korea

Telephone: 82-2-3410-3476

Fax: 82-2-3410-0040

E-mail: kmhyj111@gmail.com

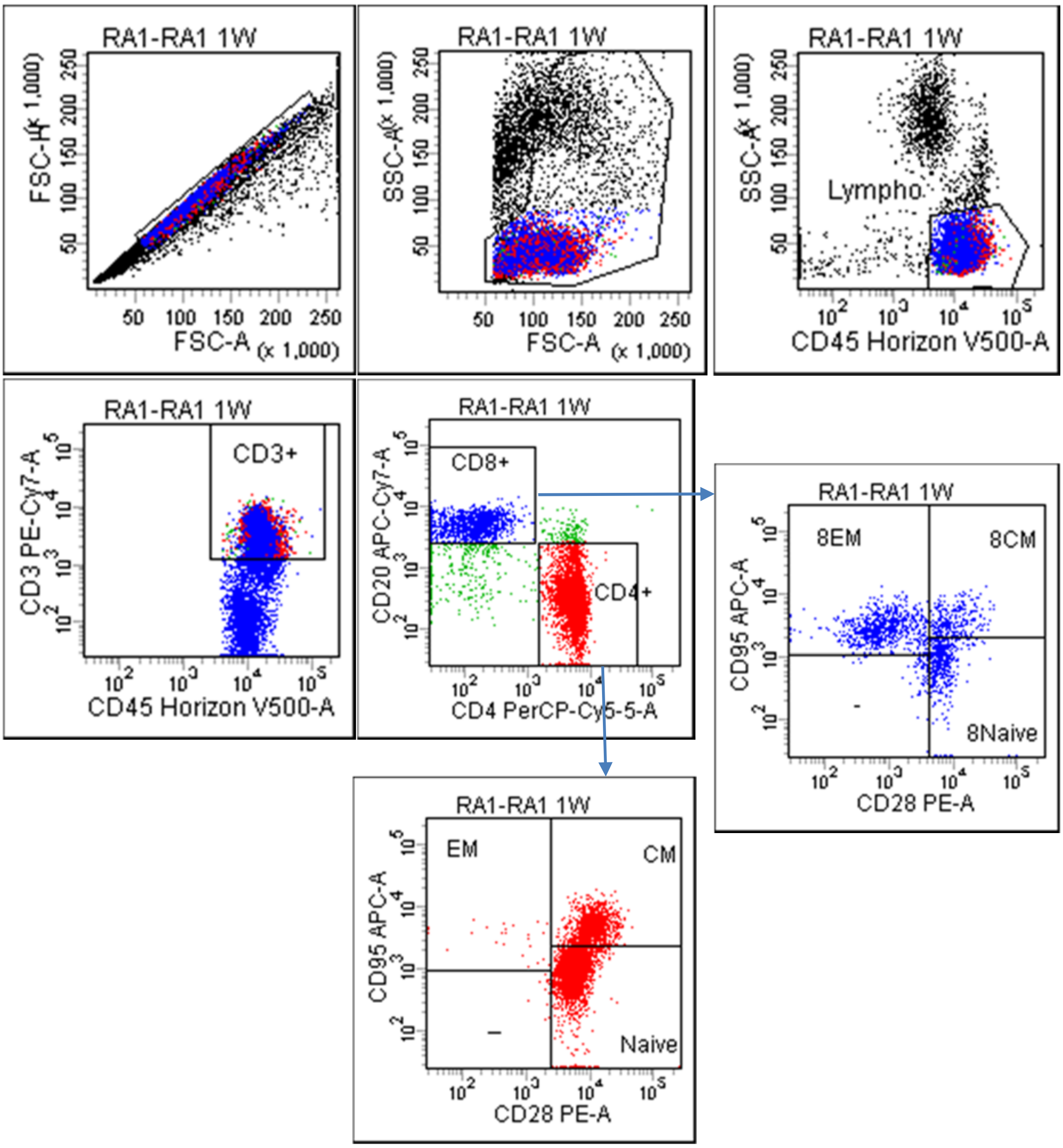

Supplementary Figure 1. A representative gating scheme of the T cell subset in the peripheral blood. For analysis of T-cell populations, peripheral blood mononuclear cells (PBMCs) were stained with V500–conjugated anti-CD45 (BD Biosciences), PE-Cy7–conjugated anti-CD3, PerCP-Cy5.5–conjugated anti-CD4, APC-Cy7–conjugated anti-CD8, PE–conjugated anti-CD28, and APC–conjugated anti-CD95 antibodies. EM: CD4 effector memory cell, CM: CD4 central memory cell, Naive: CD4 naïve T cell, 8EM: CD8 effector memory cell: 8CM: CD8 central memory cell, 8Naive: CD8 naïve T cell

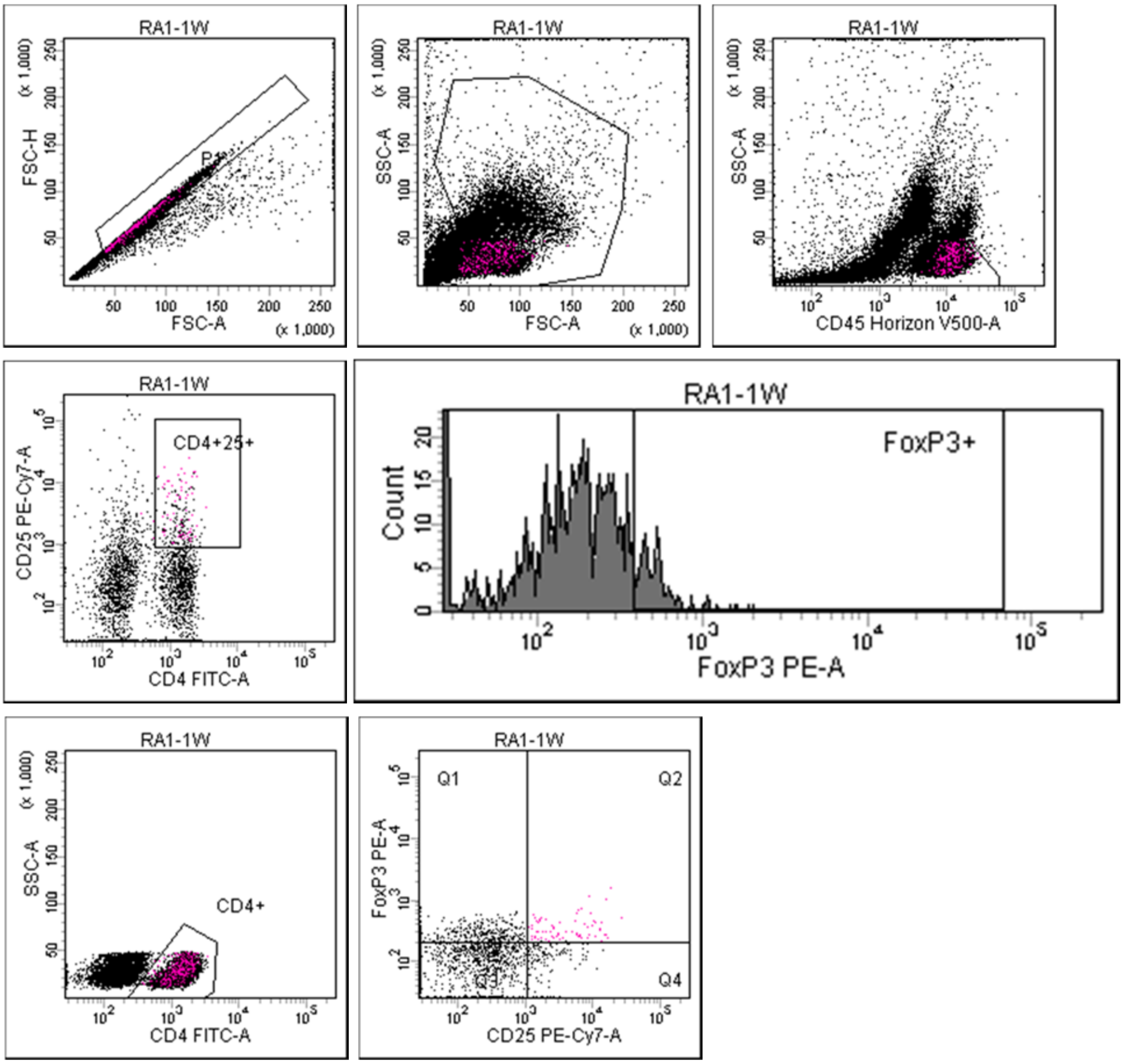

Supplementary Figure 2. A representative gating scheme of the regulatory T cells in the peripheral blood. For analysis of regulatory T cells, PBMCs were stained with V500–conjugated anti-CD45, FITC–conjugated anti-CD4, PE-Cy7–conjugated anti-CD25, and PE–conjugated anti-FoxP3 antibodies.

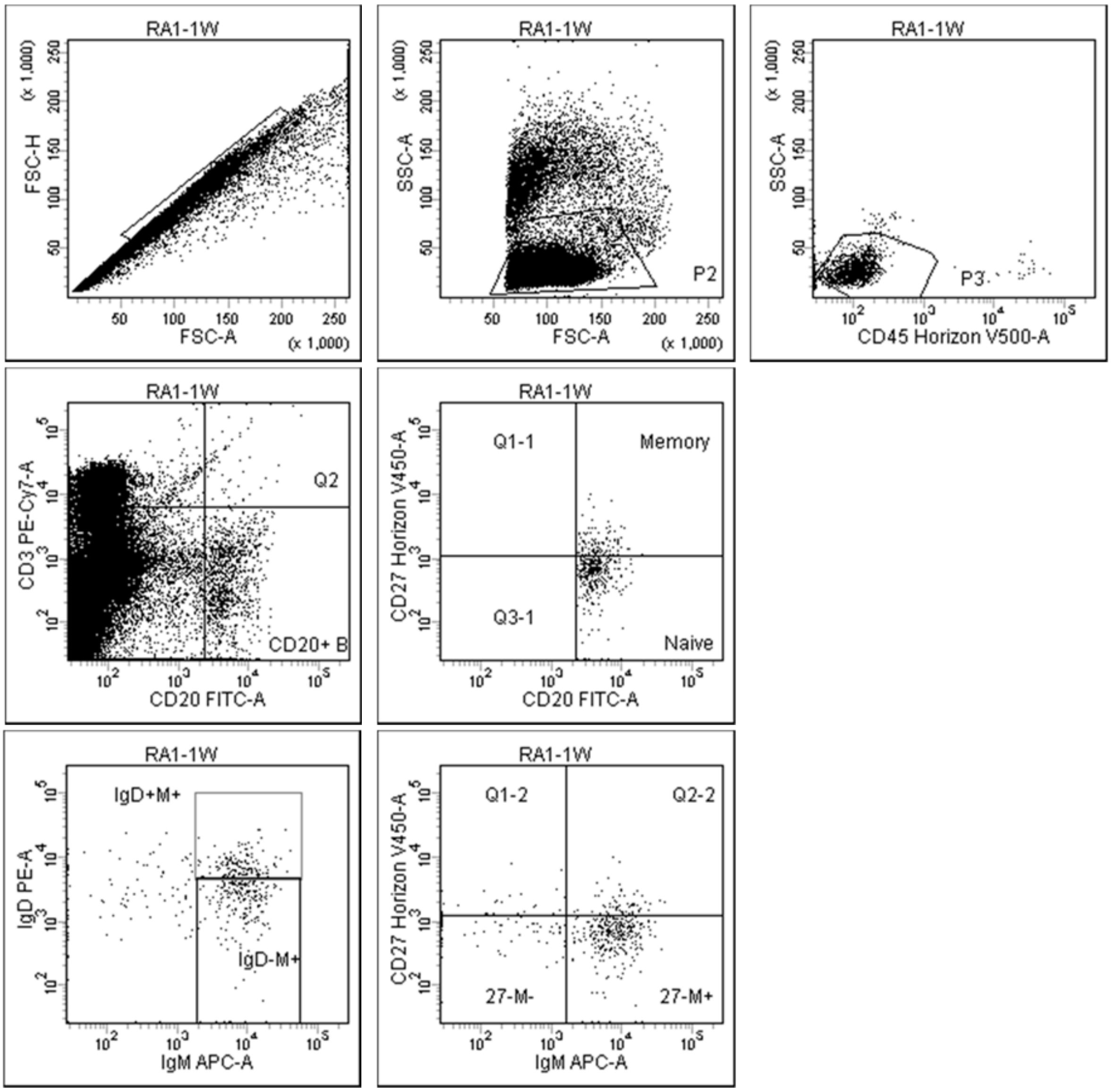

Supplementary Figure 3. A representative gating scheme of the B cell subset in the peripheral blood. For analysis of B-cell populations, PBMCs were stained with V500–conjugated CD45, PE-cy7–conjugated anti-CD3, FITC–conjugated anti-CD20, V450–conjugated anti-CD27, PerCP-Cy5.5–conjugated anti-CD21, PE–conjugated anti-IgD, and APC–conjugated anti-IgM antibodies.
